# Epinephrine in cardiac arrest: systematic review and meta-analysis

**DOI:** 10.1590/1518-8345.1317.2821

**Published:** 2016-12-08

**Authors:** Ignacio Morales-Cané, María Del Rocío Valverde-León, María Aurora Rodríguez-Borrego

**Affiliations:** 1 Instituto Maimónides de Investigación Biomédica de Córdoba (IMIBIC), Córdoba, Spain. Universidad de Córdoba, Córdoba, Spain.; 2 Universidad de Córdoba, Córdoba, Spain.; 3 Instituto Maimónides de Investigación Biomédica de Córdoba (IMIBIC), Córdoba, Spain. Universidad de Córdoba, Córdoba, Spain. Hospital Universitario Reina Sofía, Córdoba, Spain.

**Keywords:** Heart Arrest, Epinephrine, Survival, Nursing

## Abstract

**Objective:**

evaluate the effectiveness of epinephrine used during cardiac arrest and its effect on the survival rates and neurological condition.

**Method:**

systematic review of scientific literature with meta-analysis, using a random effects model. The following databases were used to research clinical trials and observational studies: Medline, Embase and Cochrane, from 2005 to 2015.

**Results:**

when the Return of Spontaneous Circulation (ROSC) with administration of epinephrine was compared with ROSC without administration, increased rates were found with administration (OR 2.02. 95% CI 1.49 to 2.75; I^2^ = 95%). Meta-analysis showed an increase in survival to discharge or 30 days after administration of epinephrine (OR 1.23; 95% IC 1.05-1.44; I^2^=83%). Stratification by shockable and non-shockable rhythms showed an increase in survival for non-shockable rhythm (OR 1.52; 95% IC 1.29-1.78; I^2^=42%). When compared with delayed administration, the administration of epinephrine within 10 minutes showed an increased survival rate (OR 2.03; 95% IC 1.77-2.32; I^2^=0%).

**Conclusion:**

administration of epinephrine appears to increase the rate of ROSC, but when compared with other therapies, no positive effect was found on survival rates of patients with favorable neurological status.

## Introduction

Cardiorespiratory arrest is the sudden and unexpected cessation of breathing and spontaneous circulation, that can be restored to a previous status, in those in whom a fatal outcome was not expected at that time^1^. This is a major problem worldwide because the incidence is estimated at around 55 out-of-hospital cardiac arrests in adults per 100,000 people, of whom only 7% survive^2^.

As regards in-hospital cardiac arrests, relevant findings in the literature were scarce, but estimates indicate that the incidence could be between 1 and 5 cases per 1.000 admissions per year, and the overall survival varies between 10% and 42%^3^.

The treatment of cardiorespiratory arrest follows recommendations published every five years, based on a number of reviews by the International Liaison Committee on Resuscitation (ILCOR), an organization formed by leading global councils and Resuscitation associations. Outstanding among them are the American Heart Association (AHA) and European Resuscitation Council (ERC). Both AHA and ERC recommendations, from 2010 and 2015, have indicated that controlled clinical trials of vasopressors vs. placebo are needed. With the present evidence, the use of epinephrine in cardiac arrest is recommended as class IIb^4^. This should be considered, as the benefits may outweigh the risks; therefore both organizations recommend the use of 1 mg of epinephrine every 3-5 min. However AHA has indicated that 40UI of vasopressin may replace the first or second dose of epinephrine^5^ ^-^ ^9^. 

Epinephrine is one of three natural catecholamines, along with norepinephrine and dopamine, that has a potent stimulatory action on α and β receptors distributed throughout the body; in the heart increases the flow speed, the heart rate and the force of contraction (chronotropic and inotropic effects on the heart) therefore increases the volume per minute, the systolic blood pressure and simultaneously the myocardial oxygen consumption. High doses produce extrasystoles and cardiac arrhythmias; they (high doses) also produce a rise in blood pressure (especially diastolic) that facilitates venous return and ventricular filling during diastole by stimulating α and β receptors. This increases total peripheral resistance, thus causing an increase in differential tension and tachycardia. If hypertension is high it can cause reflex bradycardia. Excessive and prolonged activation of the myocardium is dangerous; this could cause improper increased oxygen consumption and micro injuries that may appear in the vessels and myofibrils^10^.

Based on the foregoing discourse, the question arises about the effect of epinephrine on survival of patients suffering cardiac arrest and on the neurological status of survivors of these cardiac events. 

The objective of the review was to investigate the scientific production and evaluate the effectiveness of epinephrine in treatment of cardiac arrest in terms of survival and neurological status.

## Method 

**Figure 1:**
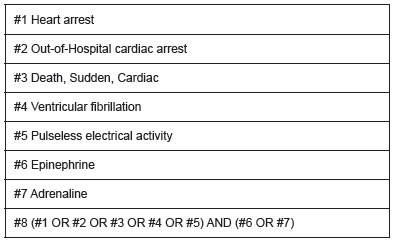
Search strategy

A systematic review of the scientific literature was performed, with meta-analysis of the results. The search was conducted in Medline, Embase and Cochrane databases, between 01/01/2005 and 02/28/2015, using free-text terms and MeSH terms: "Heart arrest", "Out-of-Hospital Cardiac Arrest", "Death, Sudden, Cardiac", "Ventricular Fibrillation", "Pulseless Electrical Activity" plus the Boolean operator "AND" with the terms "epinephrine", "adrenaline" (Figure 1). This search strategy was adapted to other databases. The full search strategy can be provided by the authors. 

### Inclusion criteria

Experimental and cohort studies, published between 01/01/2005 and 01/07/2016, evaluating the effectiveness of epinephrine in adults with cardiorespiratory arrest, compared with other therapies or without administration of a vasoactive drug.

### Outcome measures

The main outcome measure was survival to discharge/30 days. Secondary outcome measures were: survival to discharge/30 days with favorable neurologic status, one year survival, and one year survival with favorable neurologic status and return of spontaneous circulation. The neurological status was considered favorable when the patient presented score 1 or 2 on the Glasgow-Pittsburg scale "Cerebral Performance Category"(CPC)^11^ ^-^ ^12^.

### Data collection instrument

The outcomes of the bibliographic search were collected in a standardized data collection record that contained the following items:: author, year, title, purpose, hypothesis, type of study, randomization, blind, country, study duration, number of centers studied, inclusion and exclusion criteria, population, place of cardiac arrest (out-of-hospital or in-hospital) resuscitation guidelines, outcome measures, interventions, patient demographics and main outcomes.

### Descriptive and thematic analysis

The STROBE questionnaire was used to assess the quality of observational studies, and the CONSORT questionnaire to evaluate clinical trials^13^ ^-^ ^14^.

Data on clinical outcomes were grouped into short term and long term - the most common forms in cardiac arrest studies. The outcome measures were defined as follows: 1) return of spontaneous circulation, as an outcome measure in the short term and 2) survival to discharge/30 days, as an outcome measure in the long term. In the latter, the patient survived ≥30 days after cardiac arrest or left the hospital alive with favorable neurological function, obtaining a score in the categories of Glasgow-Pittsburgh brain performance of 1 (good cerebral performance) or 2 (moderate cerebral disability).

### Meta-analysis

Due to the heterogeneity of the effects contained in the studies included, data were analyzed using the Mantel-Haenszel random effects model, which was the model that best fitted for unifying the results and evaluating comparisons between epinephrine vs. no epinephrine. For other comparisons, the fixed effects model was used. A *95* % *interval of confidence (IC) was defined for both models.* Statistical heterogeneity was assessed using the I2 statistic. The cut-off scores of I^2^ ≤25%, I^2^ 26-50% and I^2^> 50% were used to define statistical heterogeneity as low, moderate, and significant, respectively ^15^. Publication bias was assessed by using funnel plots and Egger's test. Analyses were performed using the Cochrane Review Manager software (RevMan, version 5.3.5)

### Subgroup analysis

Interventions and outcome measures (when stratified data by initial heart rhythm were found) were stratified by initial rate of cardiac arrest: shockable rhythm (ventricular fibrillation and pulseless ventricular tachycardia) and non-shockable rhythm (pulseless electrical activity and asystole).

## Results

### Characteristics of studies included

**Figure 2:**
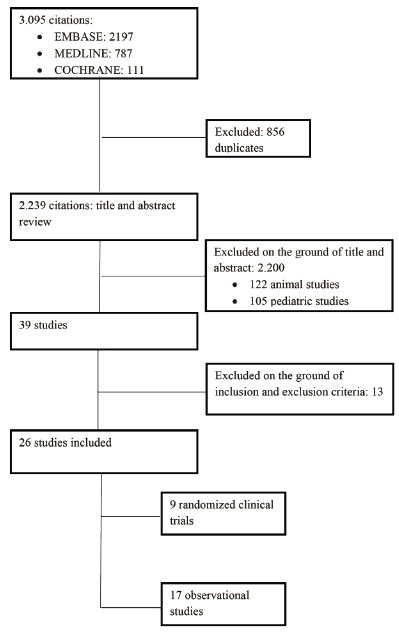
*Flow diagram of the study selection process* . 2015

After eliminating duplicate citations, 2239 references were identified. Of these, 9 randomized clinical trials and 17 observational studies were included. Details of studies selected are shown in Figure 2. 

**Figure 3:**
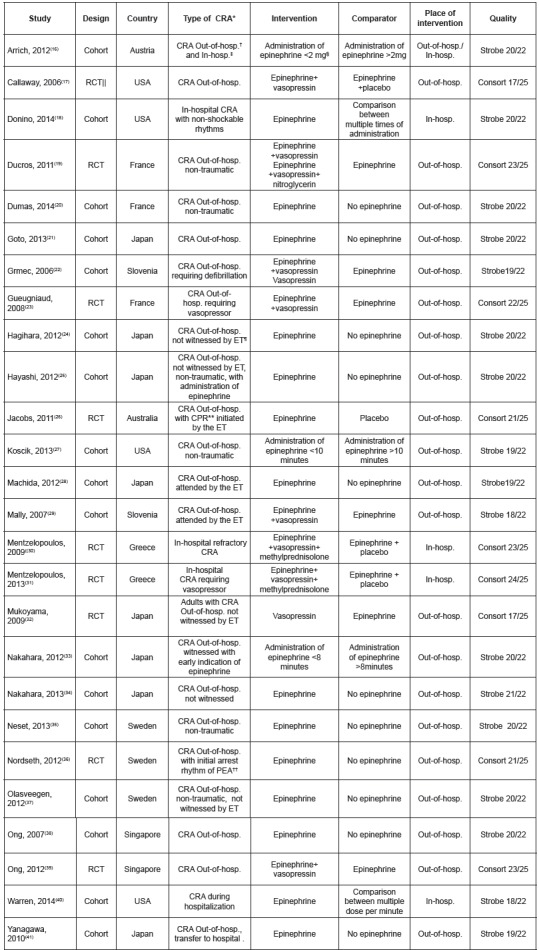
Characteristics of included studies, 2015

These studies were classified categorically, based on intervention and comparator. A summary of the characteristics of the studies included is shown in Figure 3.

*CRA: cardiorespiratory arrest; †Out-of-hosp.: out-of-hospital; ‡In-hosp.: in-hospital; §mg: milligram; ||RCT: Randomized Clinical Trial; ¶ET: emergency team; ** CPR: cardiopulmonary resuscitation; †† PEA: pulseless electrical activity

### Epinephrine vs. no epinephrine 

**Figure 4:**
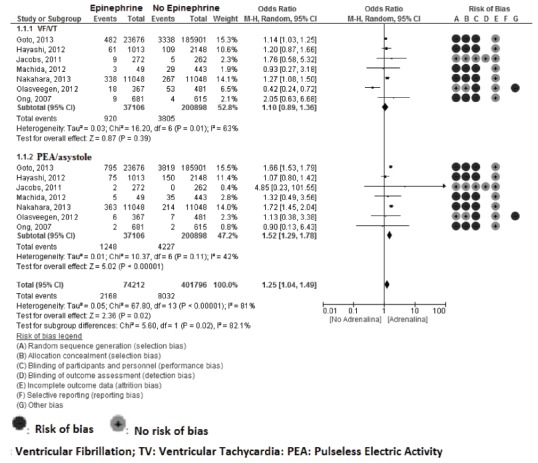
Survival to discharge/30 days. Epinephrine vs. No epinephrine stratified by FV/TV and PEA/Asystole

Ten observational studies and two clinical trials (n=655.192 patients in 12 studies) compared the administration of epinephrine with no administration of epinephrine or with placebo^20^ ^-^ ^21^ ^,^ ^24^ ^-^ ^26^ ^,^ ^28^ ^,^ ^34^ ^-^ ^38^ ^,^ ^41^. Meta-analyses showed an increase in survival to hospital discharge/30 days related to administration of epinephrine(OR 1.23; 95% IC 1.05-1.44; I^2^=83%). Stratified by ventricular fibrillation (VF) / ventricular tachycardia (VT) and pulseless electrical activity (PEA), the table shows an increase in survival to PEA/asystole (OR 1.52; 95% IC 1.29-1.78; I^2^=42%), but no significant differences were observed in VF / VT (OR 1.10; 95% IC 0.89-1.36; I^2^=63%) (Figure 4).

In the outcome survival to discharge/30 days with favorable neurologic status no significant differences were observed (OR 0.64; 95% IC 0.42-1.02; I^2^=96%). Nor were any significant stratified differences found by VF / VT (OR 0.66; 95% IC 0.29-1.51; I^2^= 98%) and PEA/asystole (OR 0.77; 95% IC 0.50-1.18; I^2^=75%).

Only one observational study^37^ ^)^ related to 1-year survival, was found, in which survival decreased with administration of epinephrine (OR 0.46; 95% IC 0.27-0.78).

Increased return of spontaneous circulation was observed with administration of epinephrine (OR 2.02; 95% IC 1.49-2.75; I^2^=95%). When stratified by VF/VT no significant differences were found (OR 1.26; 95% IC 0.90-1.76; I^2^=94%), whereas PEA/asystole showed an increase in return of spontaneous circulation with administration of epinephrine (OR 2.10; 95% IC 1.17-3.77; I^2^=99%).

### Epinephrine vs. Epinephrine + Vasopressin

Four randomized clinical trials and two observational studies ^17^ ^,^ ^19^ ^,^ ^22^ ^-^ ^23^ ^,^ ^29^ ^,^ ^39^ (n=4.358 patients included in these studies) compared the administration of epinephrine with the combination of epinephrine and vasopressin. The meta-analysis showed no significant differences in survival to hospital discharge/30 days (OR 0.94; 95% IC 0.70-1.26; I^2^=12%).

No significant differences were observed at hospital discharge/30 days with favorable neurologic status (OR 0.83; 95% IC 0.55-1.26; I^2^= 71%).

No significant differences were found in 1-year survival (OR 1.40; 95% IC 0.85-2.31; I^2^= 31%).

For 1-year survival with favorable neurologic status, only one observational study showed this outcome^39^, in which no significant differences were observed (OR 1.06; 95% IC 0.31-3.63).

In the return of spontaneous circulation, no significant differences were observed (OR 0.95; 95% IC 0.84-1.08; I^2^= 39%). Stratified by VF/VT (OR 0.94; 95% IC 0.76-1.17; I^2^=0%) and PEA/asystole (OR 0.96; 95% IC 0.58-1.56; I^2^=0%) no significant differences were observed.

### Epinephrine + placebo vs. Epinephrine + vasopressin + methylprednisolone

Two randomized clinical trials compared administration of epinephrine alone with a combination of epinephrine plus vasopressin and methylprednisolone^30^ ^-^ ^31^. The meta-analysis showed an increase in return of spontaneous circulation when epinephrine combined with vasopressin and methylprednisolone was administered (RR 1.34; 95% IC 1.18-1.52; I^2^= 37%).

### Epinephrine vs. Vasopressin

In 2 randomized clinical trials^38^ ^-^ ^39^ that compared the administration of epinephrine combined with vasopressin, no significant difference was found in survival to hospital discharge/30 days (RR 1.48; 95% IC 0.55-3.98); same outcome was found for return of spontaneous circulation (RR 1.08; 95% IC 0.76-1.53).

### Epinephrine vs. Epinephrine + vasopressin + nitroglycerin

A randomized clinical trial compared the administration of epinephrine with epinephrine combined with vasopressin and nitroglycerin. In this study there was no significant difference between the combination and epinephrine alone^19^.

### Early administration of epinephrine vs. late administration of epinephrine

Two observational studies (n=49.851 patients included in these studies) compared early administration of epinephrine (before 10 minutes) with late administration (after 10 minutes)^27^ ^,^ ^33^. 

The meta-analysis showed an increase in survival to hospital discharge/30 days for early administration (before 10 minutes) when compared with late administration (OR 2.03; 95% IC 1.77-2.32; I^2^=0%). An observational study^18^ showed a decrease in survival to hospital discharge/30 days, when epinephrine was administered in more than 9 minutes (OR 0.63; 95% IC 0.52-0.76).

### Dose of epinephrine administered

An observational study that compared different doses of epinephrine^16^ showed that administration in high doses (greater than 5.5 mg) increased hospital mortality (OR 2.82; 95% IC 1.64-4.85); and increased rates of hospital mortality and unfavorable neurological status (OR 2.95; 95% IC 1.67-5.22).

### Time between doses of epinephrine

An observational study that compared the time between doses of epinephrine during cardiac arrest^40^ observed that when doses far apart (more than 5 minutes) were compared with administration every 1-5 minutes, the former decreases the survival rate (OR 2.17; 95% IC 1.62-2.92).

The summary of results is shown in Table 1.

**Table 1:**
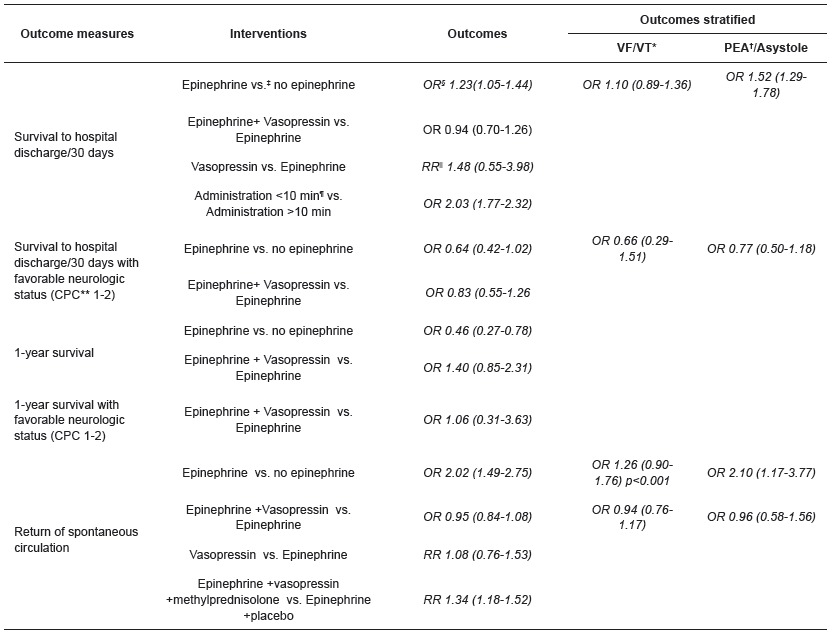
Summary of results. Spain, 2015

*VF/VT: Ventricular Fibrillation/Ventricular Tachycardia; †PEA: Pulseless Electrical Activity; ‡OR: Odds Ratio; §vs.: versus; ||RR: Relative Risk ; ¶min.: minute; **CPC: Cerebral Performance Category

## Discussion

In this systematic review and meta-analysis, a comparison was evaluated of epinephrine effectiveness during cardiac arrest with the use of other therapeutic options. 

 We did not find other comprehensive reviews and meta-analysis evaluating doses of epinephrine, time between doses, cumulative doses and short-term and long-term effects, especially in terms of neurological status. Instead we found systematic reviews and meta-analysis, in which the effect of epinephrine was compared with the use of other therapies for cardiac arrest. 

Regarding the survival to hospital discharge/30 days when epinephrine was administered in comparison with no administration of the drug, we found contradictory outcomes. The administration of epinephrine improved the rate of survival to hospital discharge/30 days^37^. However, another research^21^ stated otherwise; however, when the results were stratified by initial non-shockable rhythm the two findings coincided. 

Ong et al.^38^ stated that there were no clear benefits administering epinephrine or not, thus this author was in agreement with Dumas et al.^20^, who supported better outcomes when epinephrine was not administered. In their randomized clinical trial^39^ the combination of epinephrine and vasopressin did not increase the long-term survival rates, but they noted an increase in the number of patients arriving at the hospital admission with spontaneous circulation. On the contrary^23^, they established favorable results for the group of patients who received epinephrine as a single therapy, but the difference in comparison with the combination of epinephrine and vasopressin, was not significant.

Our findings suggested that epinephrine increased the achievement of the return of spontaneous circulation, especially when epinephrine was administered in a short period of time. This result was corroborated by other systematic reviews and meta-analysis that support the benefit of epinephrine in terms of short-term survival^42^ ^-^ ^48^.

As regards the neurological status, we found no significant differences, but the results seemed to show a decrease in rates of favorable neurological status when epinephrine was used, especially when doses were higher; However an increase in rates of favorable neurological status was found when combined vasopressin and epinephrine were administered, which was in disagreement with the systematic review with meta-analysis^45^. The authors stated that the increase in rates of favorable neurological status were higher in patients receiving standard doses of epinephrine.

We assumed that this decline in survival rates might be due to variations in heart rhythm induced by epinephrine in patients with reversed cardiac arrest, since this drug caused more instability in heart rhythm and complicated the patient's treatment^35^ ^-^ ^36^.

We could also assume that epinephrine tended to decrease the long-term survival rates, since it caused cardiac damage and increased consumption of oxygen in myocardial tissue.

Researchers found that patients treated with epinephrine for cardiac arrest and died, suffered subendocardial hemorrhage; this aspect was observed in forensic studies.

Our systematic review and meta-analysis had limitations. Firstly, most of the articles included were observational studies; therefore these uncontrolled studies included more bias in the results of the review. The second limitation was the lack of sufficient data related to some of the desired results, meaning that it was not possible to obtain statistically significant outcomes. The third limitation was the organizational structure of the emergency team and the training of its components in different countries, which introduced a bias that was not found in the articles researched.

On the other hand, most of the studies, included in this systematic review and meta-analysis, were published in high impact journals, located in the first quartile, such as: Resuscitation, The New England Journal of Medicine and The Journal of Emergency Medicine, among others.

Despite these limitations, the findings suggested that epinephrine had a positive effect on the early stages of cardiac arrest care, and favored the return of spontaneous circulation; however, it had a negative effect on survival in the short- and medium-term and on the neurological status of the patient. This suggested that the current recommendations should be reviewed, limiting the number of doses of epinephrine administered in cardiac arrest; moreover, epinephrine must be indicated in cases of cardiac arrests with a concrete cause, avoiding its administration in cardiac arrest induced by coronary syndromes, for example.

## Conclusion

The scientific evidence on the use of epinephrine in cardiorespiratory arrest was contradictory. We found epinephrine administration to be beneficial for the return of spontaneous circulation during resuscitation maneuvers; however, we did not find this benefit in the survival rate up to the time of discharge from hospital, in the long-term and on the neurological status of patients. 

We believe it is necessary to conduct high-quality studies that take into account confounding variables, such as the quality of resuscitation, route of administration and numbers of doses, with the purpose of confirming the findings based on sufficient evidence.
